# Stories of the Experience of Searching for Long-Term Care and Eldercare: Case Study and Narrative Perspectives

**DOI:** 10.1093/geroni/igaa057.248

**Published:** 2020-12-16

**Authors:** Connie Corley, Ryan McCarty

**Affiliations:** 1 Fielding Graduate University, San Gabriel, California, United States; 2 Aging Simplified, Royse City, Texas, United States

## Abstract

With over 40 million individuals aged 65 years and older in the US, and by 2050 rising to an estimated 89 million, age matters, driving an increased need for long-term care/eldercare. Coupled with the higher costs of care, the search for long-term care/eldercare services can be a difficult prospect for adult children decision-makers. We present the experiences of adult children decision-makers in the US, using two methodological approaches: narrative and case study using autoethnography. In a narrative inquiry of 9 caregivers responsible for making long-term care/eldercare decisions for their parent(s) the zoom model was applied to conduct the analysis. Findings suggest that decision-makers have a strong sense of duty towards helping their loved ones find long-term care solutions. Decision-makers searched for many types of care solutions ranging from home health care to nursing homes. The experiential response most consistently stated by the participants was stress. These results are augmented by an autoethnographic case study in “six acts” illustrating how sense of agency in the caregiving journey can be enhanced. Participants with industry experience had a minimal advantage over those with no experience when it came to navigating the search for long-term/eldercare. We highlight why stories of family search for long-term care/eldercare matter, and how they can be leveraged for fundraising, advocacy, and healing. Implications for policy, research, education and practice are highlighted.

